# Mutation spectrum of *Drosophila *CNVs revealed by breakpoint sequencing

**DOI:** 10.1186/gb-2012-13-12-r119

**Published:** 2012-12-22

**Authors:** Margarida Cardoso-Moreira, J Roman Arguello, Andrew G Clark

**Affiliations:** 1 Department of Molecular Biology and Genetics, Cornell University, 526 Campus Road, Ithaca, NY 14853-2703, USA

**Keywords:** Copy number variants, CNVs, Non-allelic homologous-recombination, NAHR, Single-strand annealing, SSA, Non-homologous end-joining, NHEJ, Replication-associated repair, Alternative end-joining, Microhomology-mediated end-joining, MMEJ, Filler DNA

## Abstract

**Background:**

The detailed study of breakpoints associated with copy number variants (CNVs) can elucidate the mutational mechanisms that generate them and the comparison of breakpoints across species can highlight differences in genomic architecture that may lead to lineage-specific differences in patterns of CNVs. Here, we provide a detailed analysis of *Drosophila *CNV breakpoints and contrast it with similar analyses recently carried out for the human genome.

**Results:**

By applying split-read methods to a total of 10x coverage of 454 shotgun sequence across nine lines of *D. melanogaster *and by re-examining a previously published dataset of CNVs detected using tiling arrays, we identified the precise breakpoints of more than 600 insertions, deletions, and duplications. Contrasting these CNVs with those found in humans showed that in both taxa CNV breakpoints fall into three classes: blunt breakpoints; simple breakpoints associated with microhomology; and breakpoints with additional nucleotides inserted/deleted and no microhomology. In both taxa CNV breakpoints are enriched with non-B DNA sequence structures, which may impair DNA replication and/or repair. However, in contrast to human genomes, non-allelic homologous-recombination (NAHR) plays a negligible role in CNV formation in *Drosophila*. In flies, non-homologous repair mechanisms are responsible for simple, recurrent, and complex CNVs, including insertions of *de novo *sequence as large as 60 bp.

**Conclusions:**

Humans and *Drosophila *differ considerably in the importance of homology-based mechanisms for the formation of CNVs, likely as a consequence of the differences in the abundance and distribution of both segmental duplications and transposable elements between the two genomes.

## Background

One of the most surprising discoveries about genome sequence variation was the finding that copy number variants (CNVs; that is, duplications, deletions, and insertions) are widespread in eukaryotic genomes. CNVs have the potential to create novel genes, to alter gene structures, and/or to change gene regulation. As a result, CNVs can cause large phenotypic effects, ranging from highly deleterious [1,2], to CNVs underlying adaptation to novel environments [3,4]. The phenotypic effects of CNVs shape their genomic distribution: in natural populations, CNVs are strongly depleted among protein-coding genes and other functional elements of the genome [5,6]. However, in addition to selection, mutational processes also impact the genomic distribution of CNVs [7-10]. The distribution of these variants is not uniform across the genome; instead, CNVs accumulate in discrete regions as a consequence of local increases in the mutation rate. Consequently, current efforts aimed at the identification of the causal CNVs of both deleterious and adaptive phenotypes could be greatly enhanced by a better understanding of the mutational processes underlying the formation of CNVs and the genomic features associated with elevated mutation rates.

CNVs are formed when the repair of DNA breaks (mostly DNA double-strand breaks) is not perfect, leading to the creation of copy-number mutations. DNA double-strand breaks arise as part of the normal metabolism of the cell or as a consequence of ionizing radiation or reactive oxygen species [11,12]. There are three molecular pathways available to repair the breaks, two that require sequence homology to perform the repair - homologous recombination (HR) and single-strand annealing (SSA) - and one that is homology-independent - non-homologous end-joining (NHEJ). Although both HR and SSA require sequence homology to repair DNA double-strand breaks, they differ in the extent of homology that is required: 100 to 200 bp for HR *versus *as little as 50 bp for SSA [11,12]. Another difference is that while SSA always creates a deletion as a consequence of the repair (it is a mutagenic repair pathway), most of the time HR repairs the DNA break without generating any mutation. However, the existence of segmental duplications (also called low copy repeats (LCRs)) or transposable elements near the DNA break can lead to misalignments in the region. In this case, the repair occurs between misaligned repeats leading to the formation of duplications and deletions in a process known as non-allelic homologous recombination (NAHR). In the absence of sequence homology the cell can use non-homologous pathways to repair DNA double-strand breaks. NHEJ, like SSA, is mutagenic, usually resulting in nucleotide substitutions or small indels, but it can also create larger insertions and deletions. While NHEJ does not require sequence homology, a related alternative end-joining pathway, microhomology-mediated end-joining (MMEJ), uses microhomology to mediate the repair [13]. The different molecular pathways are therefore associated with different types of breakpoints and classes of CNVs: NAHR is associated with large stretches of sequence identity and generates both duplications and deletions; SSA is associated with smaller stretches of sequence identity and only generates deletions; NHEJ and its associated pathways (for example, MMEJ) are associated with either presence (2 to 10 bp) or absence of microhomology, and are mostly associated with deletions and insertions (although it can also generate duplications) [11,12].

In recent years, additional molecular mechanisms have been proposed to operate in association with replication-based repair and cause CNVs. These mechanisms were proposed following the observation that a subset of human CNVs are highly complex [12,14,15]. Such complex CNVs are hard to explain given the canonical HR (and the associated NAHR) and NHEJ pathways because they would require multiple DNA double-strand breaks. Furthermore, the analysis of the breakpoints of these CNVs suggested multiple rounds of strand invasion and the copying of nearby sequences [12,14,15], signatures that could more easily be explained by replication forks stalling (or collapsing), and subsequently disengaging from the template and re-annealing. Three of the proposed models are: fork stalling and replication switching (FoSTeS) [16], microhomology-mediated break-induced replication (MMBIR) [17], and serial replication slippage (SRS) [18]. Although these models differ in specific details [12,14], they are essentially indistinguishable in terms of breakpoint analysis. They all share the requirement that the re-annealing is mediated by microhomology, and they also suggest that templated DNA from nearby sequences can be introduced at the breakpoints [12,14]. Although these models have also been proposed to mediate the formation of simple CNVs, it is challenging to distinguish the signatures of these microhomology-mediated replication models from those of NHEJ (and associated MMEJ). In principle, one could distinguish between the two when there are additional nucleotides present at CNV breakpoints: replication-based models would predict that the additional nucleotides correspond to templated DNA (that is, the extra nucleotides were copied from a nearby location) while NHEJ/MMEJ would predict that the additional nucleotides correspond to filler DNA (that is, the extra nucleotides were randomly incorporated).

Most of the work in CNV breakpoint identification has been restricted to mammalian genomes, and in particular to the human genome [19-23]. In humans (as in other mammals) CNVs are significantly enriched close to segmental duplications [8,12]. These regions were initially proposed, and subsequently shown to be, CNV hotspots predominantly through facilitating NAHR [7,8,12]. However, not all human CNV hotspots are associated with segmental duplications; in fact, a sizeable fraction is not [7,21]. Here, we aim to further our understanding of the mutational mechanisms underlying the formation of CNVs by extending breakpoint analysis to the *D*. *melanogaster *genome. CNVs are as widespread in the fly as in mammalian genomes [5,24,25], and CNV hotspots have been identified in both *D. melanogaster *[9] and its sister species, *D. simulans *[10]. Although patterns of copy number variation share many similarities between humans and flies, the two genomes have very different genomic architectures. For example, while segmental duplications comprise approximately 5 % of the human and mouse genomes, they comprise only 1% of the fly genome [26]. Similarly, while transposable elements comprise approximately 50% of the human genome, they only correspond to 20% of the fly genome, where they are mostly restricted to pericentromeric regions and the fourth chromosome [27]. (The same holds true for segmental duplications [26].) Our goal was to take advantage of the differences in genome architecture between flies and humans in order to dissect the contribution of different genomic features to the formation of CNVs. We have done this by examining two distinct sets of CNVs: one generated using long Roche/454 sequencing reads [28] and the other using high-resolution tiling microarrays [5]. The use of these two dataset sets has enabled us to overcome many of the potential biases associated with each individual method if used alone. Our results indicate that fly CNVs share several of the striking characteristics observed for human CNVs: (1) a paucity of breakpoints associated with both microhomology and additional nucleotides inserted/deleted at the breakpoints; (2) an enrichment of non-B DNA sequences at the CNV breakpoints; and (3) a significant fraction of both recurrent and complex CNVs. Importantly, however, the different architectural organization of the fly genome does appear to shape patterns of copy number variation: homology-based pathways (notably NAHR) play a minor role in the formation of fly CNVs, including recurrent CNVs. Our data indicate that in flies non-homologous pathways underlie most CNV formation for both simple and complex events. One important consequence is that in flies most insertions do not correspond to duplications of previously existing sequence but are instead created *de novo *by the random insertion of nucleotides and/or small repeats from nearby sequences.

## Results

### Precise breakpoint detection of CNVs from a 454 sequencing dataset

Sackton and colleagues sequenced at low coverage (approximately 0.2x) the genomes of nine *D. melanogaster *strains using Roche/454 technology [28]. These genome sequences were used to evaluate the extent to which population genomic inferences could be made from low/sparse genomic coverage. Sackton and colleagues identified not only SNPs, but also transposable elements and CNVs. However, the latter were identified using a paired-end framework that did not provide the exact breakpoints of the CNVs. Here, we employ a different approach to detect CNVs based on split-read mapping that is capable of detecting CNVs with precise breakpoint resolution (that is, single nucleotide resolution). Defining what is the minimum size of a variant for it to be considered a CNV as opposed to an indel is largely arbitrary and often reflects the degree of resolution of the platform used to identify those variants. While initial CNV studies defined these variants as being at least 1 kb in length, more recent studies (for example, 1000 Genomes Project [21]) use 50 bp as the lower limit for calling a variant a CNV. In agreement with the previous literature on *Drosophila *CNVs [5,10], here we use 25 bp as the lower limit to classify insertions, deletions, and duplications as CNVs.

We downloaded the raw data for the nine genomes sequenced by Sackton and colleagues [28] and aligned the reads against the *D. melanogaster *reference genome using the aligner Mosaik [29]. We discarded all reads that mapped to the reference genome and focused only on the subset of the reads that failed to map. We re-aligned these reads to the reference genome using BLAT [30] (see Methods). Because BLAT was designed to align mRNA onto genomic DNA, it does not penalize the existence of large gaps between the reads and the reference genome and provides the exact location of those gaps. By parsing the BLAT results we identified all reads that: (1) had a deletion larger than 25 bp in relation to the reference; (2) had an insertion larger than 25 bp in relation to the reference; and (3) mapped to two different locations with the 3' end of the read mapping 5' of the 5' end of the read (the pattern created by a tandem duplication). Because the nine genomes were sequenced at low coverage, our goal was not to identify all existing CNVs but instead to create a high-quality dataset of CNV breakpoints. To that effect, we applied a series of filters to minimize false-positive calls. Briefly, we required that each breakpoint was seen in at least two independent reads (from the same genome or from different genomes), that those two reads were not PCR duplicates, that the breakpoint was not located within the last 10 bp of the ends of the reads and that the breakpoint mapped to the euchromatic region of the genome. We also excluded from the dataset all deletions/insertions that corresponded to transposable element polymorphisms (that is, the deleted/inserted sequence mapped exclusively to annotated transposable elements). Finally, we identified the exact breakpoint configuration by re-aligning the reads supporting each of the breakpoints to the reference genome sequence using Clustal [31,32].

Using this pipeline, we identified 447 deletions and 197 insertions larger than 25 bp segregating in the nine genomes. Because we required that at least two independent reads supported each breakpoint we biased our sample toward CNVs segregating in multiple genomes (as opposed to being private to one of the genomes). A total of 72% of CNV calls are supported by reads from at least two of the nine genomes, with only 28% of the CNVs supported by multiple reads from the same genome. This result is expected given the sparseness of the genomic data.

We evaluated the quality of our calls by confirming a subset of these variants by PCR and Sanger sequencing. Out of 32 CNVs tested, all were confirmed by PCR and sequencing. Sanger sequencing supported not only the existence of the CNVs but also the precise breakpoint configuration. We tested an additional set of eight CNVs that were filtered out from the final dataset because the reads supporting them were potential PCR duplicates. Again, all eight CNVs were confirmed, suggesting this was a fairly conservative filter. However, because our pipeline was able to identify a large number of CNV breakpoints (*n *= 644), and because our focus is on inference of mechanisms of CNV formation from sequence patterns using high-confidence CNV calls, we favored the more conservative dataset that minimized the number of false-positives.

To investigate the existence of potential differences between the mutational mechanisms underlying the formation of insertions and deletions, we used the *D. simulans *reference genome, and a parsimony approach, to polarize the calls (see Methods). Out of 447 deletions, 338 were confirmed to be deletions segregating in the sequenced *D. melanogaster *strains, 13 were re-classified as insertions in the reference genome, and 96 could not be polarized. Out of 197 insertions, 37 were confirmed to be insertions segregating in the sequenced *D. melanogaster *strains, with 123 being re-classified as deletions in the reference genome, and 37 could not be polarized.

**Figure 1:**
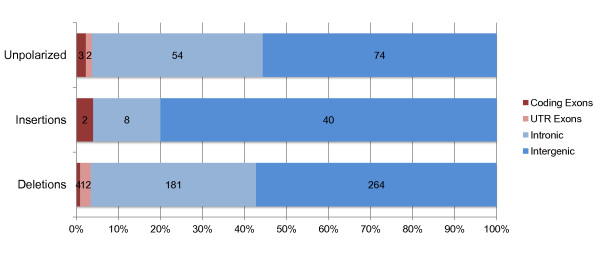
**Genomic context of the CNVs detected in this study**.

Sizes of the identified insertions and deletions ranged from 25 bp to 7.5 kb, with a median size of 34 bp and a mean size of 76 bp. The split-read method imposes no limit to the size of the deletions detected, but insertions are only detected if they are completely encompassed within a read. For this reason, the largest insertion detected in comparison to the reference genome sequence (that is, before polarization) was only 64 bp. Figure 1 shows the distribution of deletions, insertions, and unpolarized calls overlapping different functional contexts. Only nine of the 644 CNVs (1%) overlap coding exons: five are completely contained within the exon and four overlap both exonic and intronic sequence. All five CNVs located within coding exons have sizes that are multiples of three, suggesting they do not lead to frameshift mutations.

### Most insertions are not tandem duplications and correspond to *de novo *DNA

After polarization, our dataset included 50 insertions: 13 present in the reference genome sequence and 37 segregating in the strains sequenced. Of the 50 insertions, only two (4%) are tandem duplications, whereby the inserted sequence is a copy of a stretch of DNA already present in the genome (at a nearby location). Of the remaining 48 insertions, seven (14%) correspond to simple expansions of dinucleotides or small repeats flanking the insertions, and 41 (82%) have no match to the reference genome sequence and were thus classified as 'filler DNA' [13]. Filler DNA is a common outcome of the repair of DNA double-strand breaks by NHEJ in flies [33,34] and other organisms [35]. Filler DNA has been observed in several studies of DNA repair that use artificial DNA constructs where DNA double-strand breaks are induced and the products of the DNA repair can be recovered and sequenced. In most cases, only a few nucleotides (or none) are added to the repaired junctions, but in some instances large insertions are created [13,33,35].

**Figure 2:**
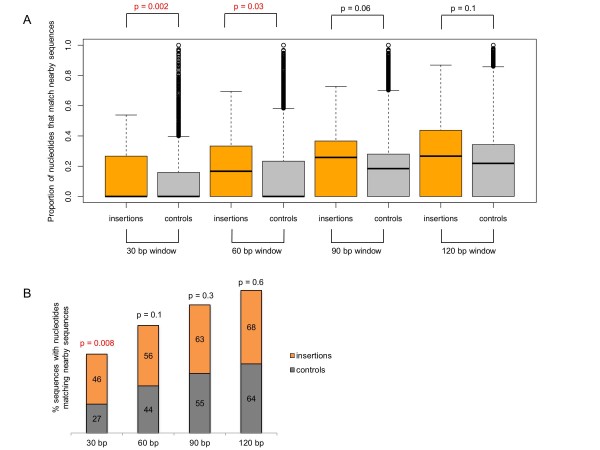
**The contribution of nearby sequences to the formation of *de novo *insertions**. (**A**) Proportion of nucleotides in insertions and matching controls that match small stretches of DNA present in nearby sequences for different window sizes (30 bp, 60 bp, 90 bp, and 120 bp windows). *P *values refer to Wilcoxon rank sum tests. (**B**) Percentage of insertions and matching controls that have at least one small stretch of DNA sequence also found in flanking regions for different window sizes (30 bp, 60 bp, 90 bp, and 120 bp windows). *P *values refer to Fisher's exact tests.

Filler DNA has been proposed to also include rearrangements of direct and inverted repeats located in nearby sequences [33]. We therefore investigated how much of each insertion classified as filler DNA could be attributed to both direct and inverted repeats present in its neighboring sequences. We considered four different window sizes to define neighboring sequences: 30 bp, 60 bp, 90 bp, and 120 bp directly upstream and downstream from the insertion breakpoints. We then quantified the number of nucleotides in the insertions that matched neighboring sequences (see Methods). We also applied this procedure to a set of 41,000 simulated insertions that we created by shuffling the genomic coordinates of the actual insertions within each chromosome (retaining the insertions sizes). The goal was to determine how much overlap between a given stretch of DNA and its neighboring sequences is expected by chance. The boxplots in Figure 2A show the distribution of the proportion of nucleotides in insertions (and nucleotides in the simulated insertions) that match neighboring sequences. For the two smallest window sizes (30 bp and 60 bp upstream and downstream from the insertions), the proportion of nucleotides in insertions that could be attributed to the copying of small stretches of DNA from neighboring sequences was significantly higher than what is expected by chance (Wilcoxon rank sum test, *P *= 0.002 and *P *= 0.03, respectively). Accordingly, there is an excess of insertions with nucleotides matching neighboring repeats over the random expectation for the smallest window size (30 bp): 46% of insertions have nucleotides that match repeats in neighboring sequences *vs*. 27% of random sequences (Fisher's exact test, *P *= 0.008; Figure 2B). When larger window sizes are considered, a much larger fraction of insertions (and of nucleotides within those insertions) matches repeats in neighboring sequences. However, this is not different from what is observed for the set of simulated insertions (Figures 2A and 2B). Importantly, the matching repeats are typically small stretches of DNA (approximately 7 to 13 bp) and so even when present, they represent only a small fraction of the total number of inserted bases.

These data suggest that most insertions in *D. melanogaster *do not correspond to tandem duplications or to expansions of di- or tri-nucleotides or repeats, but instead that they are the product of the random incorporation of nucleotides and of the copying of small stretches of DNA from nearby sequences as part of the process of DNA repair. Although anecdotal, the fact that the two tandem duplications identified are also two of the largest insertions might be interpreted as suggesting that larger insertions may indeed correspond mostly to tandem duplications while smaller insertions (that is, smaller than 60 bp) will mostly correspond to novel stretches of DNA sequence. The observation that most insertions in *Drosophila *correspond to novel DNA sequence contrasts with a previous observation made for the human genome, where most recent insertions (1 to 100 bp; appeared after the human-chimpanzee split) were determined to correspond to tandem duplications [36].

### Distinct classes of CNV breakpoints

**Figure 3:**
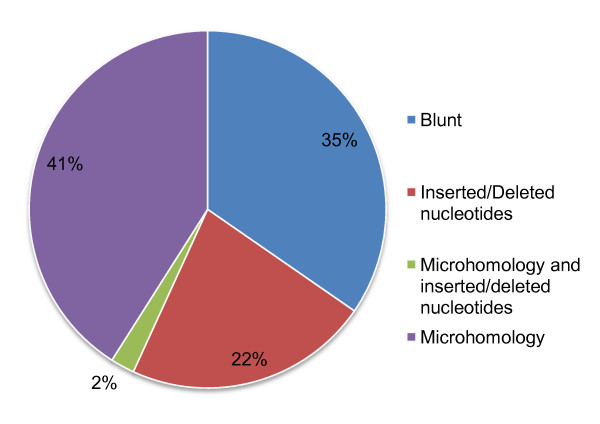
**Distribution of CNVs among the different classes of breakpoints**.

The CNVs in our dataset fall into four breakpoint classes: (1) 41% have simple ends associated with small stretches of microhomology (minimum of 2 bp); (2) 35% have blunt ends; (3) 22% have complex ends with additional nucleotides added or deleted to the breakpoint; and (4) 2% have complex ends (nucleotides added or deleted) and are also associated with stretches of microhomology (Figure 3). Microhomology is almost exclusively associated with simple ends, with only 5% of the breakpoints with microhomology also having additional inserted/deleted nucleotides at the breakpoints. This result mirrors the observations made for human CNVs where only a minority of breakpoints with microhomology also had inserted/deleted nucleotides at the breakpoint (Table 1[19,20]). There are no differences between insertions and deletions in the relative proportions of the four different types of breakpoints (Chi-square test, *P *= 0.54).

**Table 1:** Comparison of the types of CNV breakpoints identified in *Drosophila *and humans.

Type of breakpoint	*Drosophila*		**Human (Conrad *et al*.)**^a^		**Human (Kidd *et al*.)**^b^		Molecular mechanism(s)
		
	*n*	%	*n*	%	*n*	%	
Blunt	223	35	58	19	82	11	NHEJ
Microhomology	262	41	151	50	289	39	MMEJ, replication-associated repair
Blunt and large stretches of sequence identity (³20 bp)	2	0.3	3	1	219	29	SSA, NAHR, replication-associated repair
Inserted/deleted bases	143	22	81	27	153	21	NHEJ, replication-associated repair
Inserted/deleted bases and microhomology	14	2	9	3	3^c^	0.4	MMEJ, replication-associated repair
Total	644		302		743		

The dataset of Conrad *et al*. refers only to deletions while the dataset of Kidd *et al*. includes both deletions and insertions. Excluded from both datasets of human CNVs were those variants classified as VNTRs (variable number of tandem repeats) and as transposable elements insertions. Further excluded from the dataset of Conrad *et al*. were 13 deletions that were also associated with inversions. Because Conrad *et al*. required only 1 bp of identical sequence to call a breakpoint as being associated with microhomology, we re-classified the entire dataset so that only deletions associated with at least 2 bp of identical sequence at the breakpoint were classified as being associated with microhomology.

The definition of what constitutes a breakpoint associated with microhomology differs across studies with some authors requiring only 1 bp of identical sequence at the breakpoint (for example, [19]), while others require a minimum of 2 bp or more *(*for example, [20]). In order to determine the minimum number of identical nucleotides present at a breakpoint that are functionally relevant for CNV formation, we determined the number of nucleotides associated with three distinct types of microhomology for each of the 644 breakpoints in our dataset (Additional file 1, Figure S1). Microhomology of type I is the mechanistically-relevant form of microhomology associated with CNV formation: the deletion occurs between two sequences with microhomology such that one of the sequences becomes part of the deletion (the converse occurs in the case of an insertion). Microhomologies of types II and III (Additional file 1, Figure S1A) are not mechanistically associated with the formation of CNVs but can be used to determine the empirical expectation of finding a similar sequence of *n *nucleotides close to the breakpoints by chance. As shown in Additional file 1, Figure S1B, only for 2 bp or more do we find a significant excess of microhomology of type I *versus *the other two types (proportions test, *P *= 2.2 × 10^-12^). As a result, in this study we required a minimum of 2 bp of identical sequence to classify a breakpoint as showing evidence for microhomology. Of the 248 deletions associated with microhomology, only two have a stretch of microhomology >20 bp. Thus, at most only two of the deletions in our dataset could have been created by SSA. This is likely to be an over-estimate because previous work in *Drosophila *has suggested that larger stretches of sequence identity are required to mediate SSA [37]. All other CNVs associated with microhomology consequently are either the product of NHEJ, MMEJ, or of replication-associated repair.

CNV breakpoints harboring complex ends (that is, additional bases present at the breakpoint) are significantly larger than CNVs associated with blunt ends, irrespective of the presence/absence of microhomology (median size of 43 bp *vs*. 32 bp, Wilcoxon rank sum test *P *= 1 × 10^-13^). For 10 of 157 breakpoints with complex ends, the stretches of additional nucleotides inserted are large enough (*>*20 bp) that they could potentially be mapped to the genome. If replication-based repair mechanisms are involved, the sequences of inserted bases are expected to map to the genome, often close to the deleted sequences. If NHEJ (or a form of alternative end-joining) is involved, the inserted bases should correspond to randomly inserted nucleotides and/or to rearrangements of repeats from nearby sequences (as seen for most insertions). There is no good genomic sequence match for any of the stretches of inserted bases. Furthermore, for seven of the 10 breakpoints, there are small stretches of identity between the inserted bases and nearby sequences that resemble the type of alignments seen between *de novo *DNA insertions and nearby sequences. These data favor the hypothesis that these CNVs are a consequence of NHEJ or alternative end-joining repair.

Table 1 compares the types of breakpoints identified in this study with those of two previous surveys of human CNV breakpoints [19,20]. There are two main differences between the types of breakpoints observed in *Drosophila *and in humans. The first is that in *Drosophila *there is a higher proportion of blunt breakpoints, a common outcome of NHEJ (35% in *Drosophila vs*. 11% to 19% in humans). The second is that in *Drosophila *breakpoints are rarely associated with large stretches of high sequence identity, the hallmark of SSA and NAHR, while in humans Kidd and colleagues found that almost one-third of all breakpoints bore the hallmarks of these pathways [20]. As is clear from Table 1, the two surveys of human CNVs found a very different proportion of breakpoints potentially associated with NAHR (1% in Conrad *et al*. *vs*. 29% in Kidd *et al*.). This difference is likely a consequence of the different experimental approaches used between the studies. Conrad and colleagues used a microarray capture strategy to identify the breakpoints of a subset of CNVs identified in a previous study [6], which may have biased their sample against CNVs associated with NAHR. Kidd and colleagues, on the other hand, identified CNV breakpoints using capillary sequencing of fosmid clone inserts, a powerful approach to sample the full spectrum of CNVs. Further support for a sizeable portion of human CNVs being associated with NAHR, comes from two other studies: one estimated that approximately 28% of breakpoints are associated with NAHR [22] and the other put it closer to 10% to 15% [6]. Motivated by the observation that different technological approaches can produce different results regarding the role played by NAHR in the formation of CNVs, we decided to re-analyze a dataset of 3,639 *Drosophila *CNVs identified using high-resolution tiling arrays [5] and determine if the observation that there is a paucity of *Drosophila *CNVs associated with NAHR is robust to the CNV detection platform used.

### NAHR plays a minor role in the formation of CNVs in *Drosophila*

Emerson and colleagues [5] used tiling arrays covering the *Drosophila *genome at a resolution of 36 bp to identify 3,639 CNVs (2,211 duplications and 1,428 deletions) segregating in the genomes of 15 worldwide strains of *D. melanogaster*. Microarrays can only probe unique regions of the genome (that is, the probes in the microarray have to map to a unique genomic location), which means that they are biased against detecting additional duplications of regions of the genome that have already been recently duplicated. However, they are unbiased at detecting duplications of unique sequence where copy number changes from one copy (two copies in a diploid genome) to two copies (three or four copies in a diploid genome depending on the duplication being homozygous or heterozygous), irrespective of the presence/absence of flanking duplications. Therefore, we examined the breakpoints of these 3,639 CNVs for the presence of stretches of high-sequence identity in order to determine the contribution of homology-based mechanisms (such as SSA and NAHR) to the formation of CNVs in *Drosophila*.

Unlike the 454 data, microarray data do not provide the exact breakpoint location. As a consequence, to look for the presence of stretches of high-sequence identity we considered the sequences 500 bp upstream and downstream the predicted CNV breakpoint and the CNV sequence itself. We looked for two types of sequence homology: (1) stretches at least 30 bp in size with a sequence identity of at least 98% (type I; hallmark of SSA); and (2) stretches at least 200 bp in size with a sequence identity of at least 95% (type II; hallmark of both NAHR and SSA) (see Methods).

We found that only 2% (74/3639) of all CNVs were associated with sequence homology of type I (capable of mediating SSA), and 2.6% (95/3639) with sequence homology of type II (capable of mediating SSA or NAHR). Because deletions in this dataset were associated with a high false-positive rate (47%), we also restricted these analyses only to duplications (false-positive rate of 14%). Among the set of duplications, only 2.1% (46/2211) are associated with sequence homology type I, and 2.3% (51/2211) with sequence homology of type II. Therefore, these results support the observation made using the 454 reads that homology-based mechanisms (SSA and NAHR) play a very limited role in the formation of CNVs in *Drosophila*.

Because both next generation sequencing and microarray technologies are biased against the detection of CNVs in non-unique regions of the genome (that is, segmental duplications and transposable elements) inferences about the importance of homology-based mechanisms are necessarily restricted to unique regions of the genome. However, unlike the human genome where segmental duplications and transposable elements can be found throughout the euchromatin, in *Drosophila *most repetitive elements are confined to the regions surrounding the centromeres (which have very low rates of recombination) with only a minority of these elements present in regions of the euchromatin with normal recombination rates [26,27]. Hence, our work suggests that outside of pericentromeric and telomeric regions, homology-based mechanisms play a minor role in CNV formation in *Drosophila*.

### Very high rate of CNV recurrence in *Drosophila*

CNVs are classified as recurrent when different individuals carry independent but overlapping CNVs. The proportion of recurrent CNVs in the human genome has been estimated to be between 6% and 29% [6]. The sparseness of the 454 dataset prevents us from estimating from these data the proportion of recurrent CNVs in *Drosophila*. Therefore, in order to evaluate whether CNV recurrence is a common phenomenon in this taxon, we selected 26 genomic regions known to harbor at least one deletion in at least two strains based on the high-resolution tiling array dataset, and screened them in 15 worldwide strains by PCR and Sanger sequencing. These deletions are all located in the euchromatin, their mean size is similar to the mean size of the whole set of deletions and were predicted to range in frequency from 2 to 11 (median 2). Among the 26 regions, 12 (46%) harbored more than one overlapping CNV, suggesting a high rate of CNV recurrence in *Drosophila*.

Sanger sequencing of these 26 regions showed that the CNVs identified with the tiling arrays have identical characteristics to those identified with the 454 reads. There is no difference in the distribution of breakpoints types present in the 454 dataset and in the set of 36 CNVs (33 deletions and three insertions) segregating in the 26 regions described above (a total of 42 CNVs were detected but for six (mostly tandem duplications) the breakpoints were not fully sequenced). In addition, similar to what was observed in the 454 dataset, CNVs with breakpoints harboring additional bases were, on average, larger than CNVs with simple breakpoints (that is, blunt ends with or without microhomology) (median 432 bp *vs*. 211 bp, respectively; Wilcoxon rank sum test, *P *= 0.005).

There was no difference in the distribution of breakpoint types between recurrent and non-recurrent CNVs. Furthermore, just as seen for the non-recurrent set, the recurrent CNVs were not associated with large stretches of sequence identity that might suggest their generation through NAHR. Instead, these data suggest that recurrent CNVs are mediated by non-homologous repair mechanisms. Among the 12 regions showing recurrent CNVs, three also show evidence for the presence of complex CNVs. These occur when a single mutational event generates several breakpoints, that is multiple closely located CNVs segregating within the same individual. In these three regions the distance between distinct breakpoints ranged from 82 bp to 325 bp. This association between complex CNVs (multiple CNVs within the same individual) and recurrent CNVs (multiple CNVs segregating in different individuals) suggests that some regions of the *Drosophila *genome are particularly unstable, and generate both complex events within individuals as well as independent but overlapping mutations between individuals. Though the sample size is small, these data suggest that complex CNVs may correspond to as much as 12% (3/26) of all *Drosophila *CNVs, a higher proportion than the 5% estimated for the human genome [6].

### Non-B DNA structures are enriched in CNV breakpoints

DNA conformations that do not correspond to the right-handed Watson-Crick double-helix are collectively termed non-B DNA [38,39]. These include sequences with Z-DNA motifs, quadruplex-forming motifs, inverted repeats, mirror repeats, and direct repeats [40]. Non-B DNA sequences have been found associated with the causal variants of several human diseases and have been proposed to cause genetic instability by impairing DNA repair and DNA replication [38,39]. Because errors during DNA repair and DNA replication are the ultimate causes of CNVs, we tested for the presence of non-B DNA sequence at the CNV breakpoints identified using the 454 data.

**Figure 4:**
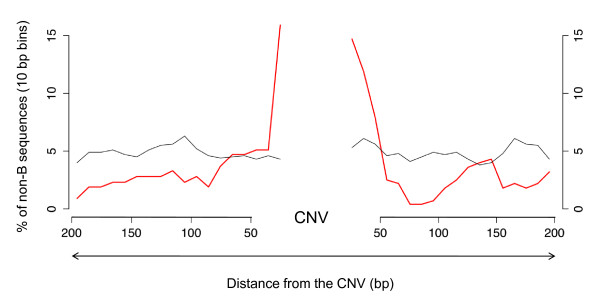
**Distribution of non-B DNA sequences in the regions surrounding CNVs**. In gray is the background expectation (determined from 4,470 control sequences) for the presence of non-B DNA sequences in a given stretch of DNA and in red the actual distribution of non-B DNA sequences surrounding the set of CNVs identified in the 454 dataset.

We focused on those variants that were detected by the presence of gaps in the reads of the sequenced genomes in comparison to the reference genome (*n *= 447) so that we could extract the CNV region and the flanking regions directly from the reference genome. For a control dataset, we shuffled the coordinates of the CNV breakpoints (25 bp within the CNV and the 200 bp immediately flanking it 5' and 3') randomly within chromosomes, so that there were 10 times more control sequences than CNV breakpoints. For both CNV breakpoints and control sequences, we identified non-B DNA sequences using the non-B DNA Motif Search Tool [40]. Figure 4 shows the distribution of non-B DNA sequences across the 200 bp flanking the CNVs. In strong contrast to control sequences (in grey), which show a uniform distribution of non-B DNA sequences across their length, CNVs (in red) are enriched with non-B DNA sequences precisely at the breakpoints. Furthermore, there is a significantly higher number of CNV breakpoints (defined as the region spanning 25 bp within the CNV and the 25 bp immediately flanking it) associated with non-B DNA structures when compared to the control sequences: 11% *vs*. 5% (Fisher's exact test, *P *= 1.3 × 10^-5^).

Some classes of non-B DNA sequences are more common than others (in both CNVs and control sequences), but for most we found a shift in the location of these repeats/motifs towards the CNV breakpoint when compared to the control sequences (Additional file 2, Figure S2), suggesting that most classes of non-B DNA sequences are associated with CNV formation. We found the non-B DNA sequences equally associated with the three classes of breakpoints (that is blunt breakpoints, breakpoints associated with microhomology, and breakpoints containing additional nucleotides inserted or deleted; Fisher's exact test, *P *= 0.98). However, we found a significantly higher proportion of insertions associated with non-B DNA sequences than deletions (Fisher's exact test, *P *= 0.002). The presence of non-B DNA sequences at a significant fraction of CNV breakpoints suggests a potential causal role for these sequences in CNV formation in flies.

## Discussion

The detailed analysis of *Drosophila *CNV breakpoints suggests that non-homologous repair mechanisms are responsible for the formation of the majority of the variants. This is true not only for simple CNVs, but also for those that are recurrent and complex. We excluded a significant role for homology-based pathways (that is, NAHR and SSA) in the formation of CNVs because only a minority of these variants (approximately 3%) are flanked by stretches of high sequence identity. We also found little support for replication-associated mechanisms; the large stretches of additional nucleotides present at 10 breakpoints consisted of filler DNA, a result more consistent with NHEJ than with replication-associated repair. The presence of microhomology at CNV breakpoints is, however, consistent with NHEJ, MMEJ, and replication-associated repair (for example, [41]). Determining exactly which pathway(s) are responsible for the different types of CNV breakpoints identified in our study will require the analysis of CNV breakpoints from fly mutants lacking the specific genetic requirements for each pathway (for example, [42,43]).

In the human and mouse genomes, NHEJ/MMEJ also underlie a large fraction of CNVs, though a sizeable fraction of CNVs are also mediated by NAHR (approximately 18% to 35%) [19-22,44]. The difference in the preponderance of NHEJ/MMEJ in flies and mammalian genomes does not have to reflect intrinsic differences between these taxa in the relative usage of the different repair pathways (HR *vs*. NHEJ). In fact, NAHR and SSA are highly efficient in repairing DNA double-strand breaks in flies when these occur in artificial constructs flanked by repeats that can mediate these pathways [37]. Instead, the difference we observe between the taxa in the preponderance of homology-based mechanisms to the formation of CNVs likely reflects the different genomic architectures of the genomes: abundant and widespread presence of segmental duplications and transposable elements throughout mammalian genomes and less abundant and more restricted location (to pericentromeric and telomeric regions) of these elements in the *Drosophila *genome.

The *Drosophila *CNVs used in this work are significantly smaller than the published human CNVs. As a consequence, there is the possibility that some of the differences found between the two taxa may reflect different mutational mechanisms operating on CNVs of different size. We note, however, that in flies we found no differences between the breakpoints identified using the 454 data and those found using the high-resolution tiling array data despite the fact that the latter are significantly larger. Although the CNVs identified with the high-resolution tiling arrays are still smaller than those identified for the human genome, their size range already shows a significant overlap with that of the human CNVs used in this study. The absence of large CNVs in the fly genome likely reflects the much higher compactness of this genome (a much higher gene density means that large CNVs would overlap multiple genes) and the greater strength of purifying selection.

We have attempted to circumvent technical biases in CNV detection by making sure that our observations were robust to the platforms used to identify CNVs (that is, both next generation sequencing and hybridization based platforms). Still, our conclusions have to be necessarily restricted to the euchromatic sequence located outside of both pericentromeric and telomeric regions. The latter are highly enriched with transposable elements and segmental duplications making CNV detection extremely challenging irrespective of the platform used. The analysis of the CNV data generated by tiling arrays suggests both a high level of CNV recurrence and complexity (and coupling between the two) that can only be fully explored with technologies that are not biased against the detection of these classes of variants. The development of strobe sequencing technology and of ever larger reads [44] will greatly enhance this effort. By collecting a large number of breakpoints from a sparse low-coverage genomic dataset, we have demonstrated that the analysis of CNV breakpoints does not depend on high coverage datasets, instead read size is likely to matter the most.

Despite a minor role for NAHR, the fly genome is still punctuated by CNV hotspots [9,10], that is, regions of the genome experiencing higher CNV mutation rates. These hotspots may share the properties of the mammalian CNV hotspots that are not associated with NAHR [7,45]. Unlike NAHR-mediated hotspots, where the reason for genomic instability is relatively well understood (that is, the presence of repeats leads to misalignments between homologous regions during DNA repair leading to further duplications/deletions), it is not known what causes the instability associated with the remaining hotspots. One possibility is that these correspond to regions more prone to DNA breaks and/or that are harder to repair. In support of this hypothesis we do find that non-B DNA sequences, which are capable of impairing both DNA replication and DNA repair, are significantly enriched at CNV breakpoints.

One of the most surprising results stemming from this work is that in *Drosophila *most insertions correspond to *de novo *sequences. These novel stretches of DNA sequence are as large as 60 bp in our dataset, potentially translating into the addition of 20 novel amino acids to a protein sequence. While it is likely that most of these insertions are deleterious, the occasional beneficial mutation could dramatically change the protein sequence in one single mutational event. This would allow very fast protein sequence evolution between closely related species. The frequent creation of novel stretches of DNA sequence as a consequence of DNA repair could have implications for the generation of genetic novelties and genome evolution in general.

## Conclusions

Our results suggest that the different architectural features of the *Drosophila *and human genomes shape the mutation processes responsible for generating duplications, deletions, and insertions. Homology-based pathways contribute significantly more to the formation of CNVs in humans than *Drosophila *because of the abundance and widespread presence of segmental duplications and transposable elements in humans that can mediate HR. Instead, non-homologous repair is responsible for most CNVs in flies, including complex and recurrent CNVs. Non-homologous repair is also responsible for the creation of insertions made of *de novo *sequence, which have the potential to mediate rapid protein evolution. In addition, we show that non-B DNA sequences are enriched at CNV breakpoints, which makes these sequences good candidates for being associated with regions of higher CNV instability.

## Methods

### Detection of CNV breakpoints in the 454 data

Split-read methods were first applied to long Sanger sequencing reads [45] and CNVs were detected by identifying those reads which, when mapped to the reference genome exhibit a 'split' signature, either a gap in the reference genome (which suggests an insertion in the read, Additional file 3, Figure S3), a gap in the read (which suggests a deletion in the read, Additional file 3, Figure S3), or two sections of the read mapping to the genome with their positions flipped (which suggests a tandem duplication). Until recently, split-read methods were not widely used because of the small size of the reads produced by next generation sequencing platforms. Roche/454 technology is capable of generating reads >100 bp in size, however eukaryotic genome sequencing projects have predominantly relied on Illumina reads, which only recently achieved the 100 bp mark [46]. With these longer reads, split-read methods can readily identify with precise resolution the breakpoints of duplications, deletions, insertions, inversions, and translocations, as recently shown by the 1000 genomes project [21].

Sackton and colleagues used 454 technology to sequence at low coverage (approximately 0.2x) the genomes of nine *D. melanogaster *strains (three from an African population and six from a North Carolina population) [28]. We downloaded the original 454 reads (mean read size of 105 bp) from the Short Read Archive (SRP001156) and aligned them to the release 5 of the *D. melanogaster *genome using Mosaik (version 1.1.0021) [29]. We used the following Mosaik parameters to conduct the alignments: -hs 15 -mmp 0.05 -mhp 100 -act 26 -p 8 -bw 51. We discarded all reads that Mosaik mapped to the genome and kept only those that could not be mapped. We then used BLAT (version 3.4) [30] to map the latter reads. We ran BLAT using two sets of parameters: -fastMap and -oneOff==1. Finally, we detected CNV breakpoints with custom Perl scripts that parse the BLAT output and identify the split-read signature detailed in the Results section.

As discussed in the Results section, we applied a series of filters in an attempt to minimize the number of false-positive calls. One of the filters applied was the removal of all CNVs supported exclusively by reads that could be PCR duplicates; these were defined as reads with the same exact start position but that could vary in their end position. Because of this filter all seven putative tandem duplications identified by our pipeline were excluded from the final CNV dataset. Another filter was the exclusion of all CNVs where at least 80% of the mutated sequence mapped to a known TE (TE annotation downloaded from FlyBase [47] release 5.29). After CNVs were identified, we re-aligned all supporting reads once again to the reference *D. melanogaster *genome using Clustal [31,32] and those were the alignments used to classify the CNVs into the four classes of breakpoints. The CNV calls were polarized using the syntenic alignments between *D. melanogaster *and *D. simulans *[47]. If a deletion is called in one of the nine sequenced genomes but is also present (with similar breakpoints) in the *D. simulans *genome, then the most parsimonious explanation is that the variant is actually an insertion in the reference *D. melanogaster *genome (Additional file 3, Figure S3). Similarly, if an insertion is called in one of the nine genomes but a similar insertion is found in *D. simulans*, then the most parsimonious explanation is that we are detecting a deletion in the reference *D. melanogaster *genome (Additional file 3, Figure S3). CNVs were annotated (as exonic, intronic, and intergenic) using release 5.33 (retrieved from FlyBase [47]).

### Evaluating the contribution of nearby sequences to the formation of *de novo *insertions

We used standalone Blast [48] (ncbi-blast-2.2.25) to identify stretches of high sequence identity between *de-novo *insertions and its neighboring sequences (for the window sizes defined in Results). We generated the random control sequences using BEDTools (shuffleBed; version 2.13.3) [49].

### Comparison of CNV breakpoints identified in *Drosophila *and human genomes

Table 1 compares the types of CNV breakpoints identified in *Drosophila *and in two independent human datasets: one generated by Conrad and colleagues [19] and the other by Kidd and colleagues [20]. Both surveys of human CNVs included the identification of small tandem repeats (variable number tandem repeat (VNTR)) and of variants associated with the movement of transposable elements. Because studying these variants was not an aim of this work, Table 1 only refers to breakpoints of deletions and insertions. We also excluded from the dataset generated by Conrad and colleagues the 13 deletions (out of 315) that were also associated with inversions. Finally, as discussed in the Results section we only consider microhomology when there are at least 2 bp of identical sequence present at the breakpoint. That meant re-classifying the breakpoints identified by Conrad and colleagues because they only required 1 bp to classify a CNV breakpoint as being associated with microhomology (detailed breakpoint information was made available by the authors as Supplementary Material) [19].

### Evaluating the roles of NAHR and SSA

To test for sequence identity shared between regions within CNV coordinates and flanking DNA, three sequence databases were generated for both the 454 and microarray data, using the reference *Drosophila *genome (version 5.27): (1) CNV sequence; (2) 5' flanking sequence; (3) 3' flanking sequence. The 454 data provide precise CNV breakpoints and, based on these coordinates, we extracted 200 bp 5' and 3' of the CNV. The microarray data do not provide exact breakpoints, and for these data we defined the distal ends of the flanking sequences to be 500 bp 5' or 3' of the CNV coordinates. The proximal coordinates of the flanking sequences were set to extend 25% the length of the CNV 3' of the start of the CNV, or 25% the length of the CNV 5' of the end of the CNV. BLAT [30] (blatSuite.34) was used to search for sequence identity between: (1) 5' flanking sequence and the DNA within the CNV coordinates; (2) 3' flanking sequence and the DNA within the CNV coordinates; and (3) 5' flanking sequence and 3' flanking sequence. The data were filtered to return two datasets for each of these searches. The first filter was set to accept stretches of ≥30 bp that possessed ≥98% sequence identity; the second was set to accept stretches of ≥200 bp that possessed ≥95% sequence identity. The microarray results were further filtered to remove all 'self-hits' that resulted from the flanking sequences overlapping the CNV coordinates. Fasta files were generated for all sequences meeting the above criteria and were screened for repetitive sequences using RepeatMasker [50] (settings: abblast search engine, default speed/sensitivity, *D. melanogaster *annotations).

### Identification of recurrent and complex CNVs in the tiling array data

We randomly selected 26 regions of the *D. melanogaster *genome that were identified by Emerson and colleagues as having deletions and that had been confirmed by PCR [5]. We screened these 26 regions in the same 15 natural populations analyzed by Emerson and colleagues. We identified recurrent CNVs by the presence of bands of different size (generated using the same pairs of primers) in different populations. We then sequenced these different bands by Sanger sequencing.

All statistical analyses were done using the statistical package R [51] and the application Rstudio.

### Data availability

The Sanger sequences of the breakpoints of simple and complex CNVs initially identified using the tiling array data have been deposited in GenBank (KC138560-KC138678).

## List of abbreviations

CNVs: Copy number variants; FoSTeS: Fork stalling and template switching; HR: Homologous recombination; LCRs: Low-copy repeats; MMBIR: Microhomology-mediated break-induced replication; MMEJ: Microhomology-mediated end-joining; NAHR: Non-allelic homologous-recombination; NHEJ: Non-homologous end-joining; SRS: Serial replication slippage; SSA: Single-strand annealing; VNTRs: Variable number of tandem repeats.

## Competing interests

The authors confirm that they have no competing interests in the conduct of this research or preparation of this paper.

## Authors' contributions

All authors read and approved the final manuscript. MC-M, JRA, and AGC designed the study. MC-M carried most of the analyses with contributions from JRA. MC-M wrote the paper with contributions from JRA and AGC.

## Supplementary Material

Supplementary Figure 1 **Evaluation of the minimum number of identical nucleotides present at the breakpoint that is required for microhomology-mediated CNV formation**. (**A**) Schematic representation of the different classes of microhomology (type I refers to the mechanistically relevant form of microhomology associated with CNV formation). (**B**) Number of breakpoints showing *n *identical nucleotides for the three classes of microhomology. Click here for file

Supplementary Figure 2 **Distribution of non-B DNA motifs in relation to CNV breakpoints**. The beanplots in orange refer to distribution of non-B DNA repeats in the sequences flanking the CNV breakpoints (combines upstream and downstream sequences) while the beanplots in grey refer to control sequences. The red line marks the location of the CNV breakpoint (at position 25 bp of 225 bp of total sequence). Small lines refer to individual observations (control sequences have 10x more data) while the longer black line refers to the average of the distribution. Each beanplot refers to a specific type of non-B DNA motif. Click here for file

Supplementary Figure 3 **Description of the split-read approach used to detect deletions and insertions and the rational for polarizing the CNV calls**. Click here for file
