# Supplementary figures and images for: Mutation spectrum of Drosophila CNVs revealed by breakpoint sequencing
Source: Genome Biol. 2012 Dec 22;13(12):R119. doi: 10.1186/gb-2012-13-12-r119 (PMC4056370; doi:10.1186/gb-2012-13-12-r119)

## Slide 1
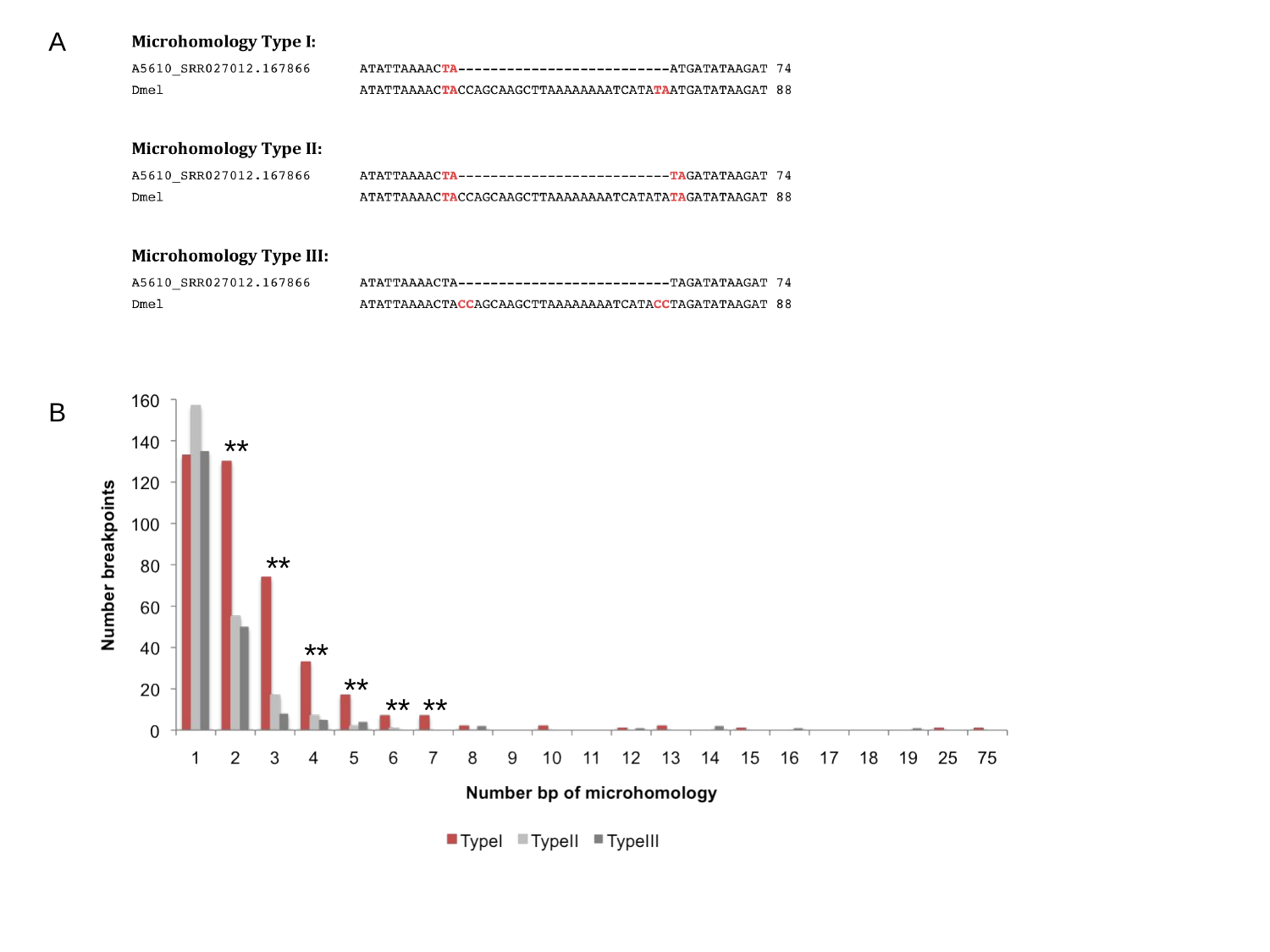

A
B
**
**
**
**
**
**

Supplement: Supplementary Figure 1 — Evaluation of the minimum number of identical nucleotides present at the breakpoint that is required for microhomology-mediated CNV formation. (A) Schematic representation of the different classes of microhomology (type I refers to the mechanistically relevant form of microhomology associated with CNV formation). (B) Number of breakpoints showing n identical nucleotides for the three classes of microhomology. [file gb-2012-13-12-r119-S1.PPT]

## Slide 1
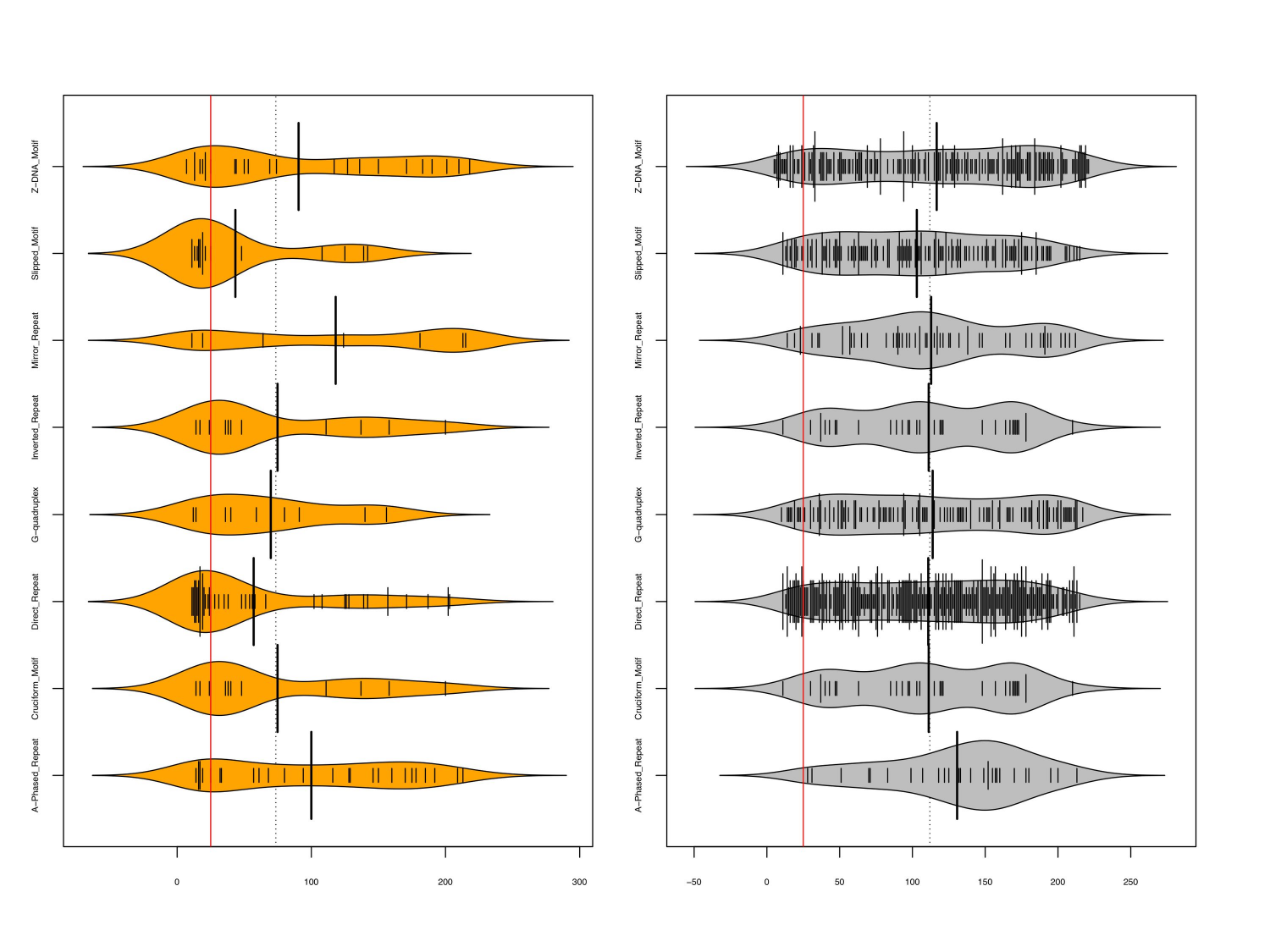

Supplement: Supplementary Figure 2 — Distribution of non-B DNA motifs in relation to CNV breakpoints. The beanplots in orange refer to distribution of non-B DNA repeats in the sequences flanking the CNV breakpoints (combines upstream and downstream sequences) while the beanplots in grey refer to control sequences. The red line marks the location of the CNV breakpoint (at position 25 bp of 225 bp of total sequence). Small lines refer to individual observations (control sequences have 10x more data) while the longer black line refers to the average of the distribution. Each beanplot refers to a specific type of non-B DNA motif. [file gb-2012-13-12-r119-S2.PPT]
