# Supplementary material for: Mutation spectrum of Drosophila CNVs revealed by breakpoint sequencing
Source: Genome Biol. 2012 Dec 22;13(12):R119. doi: 10.1186/gb-2012-13-12-r119 (PMC4056370; doi:10.1186/gb-2012-13-12-r119)
Supplement: Supplementary Figure 3 — Description of the split-read approach used to detect deletions and insertions and the rational for polarizing the CNV calls. [file gb-2012-13-12-r119-S3.PPT]

## Slide 1
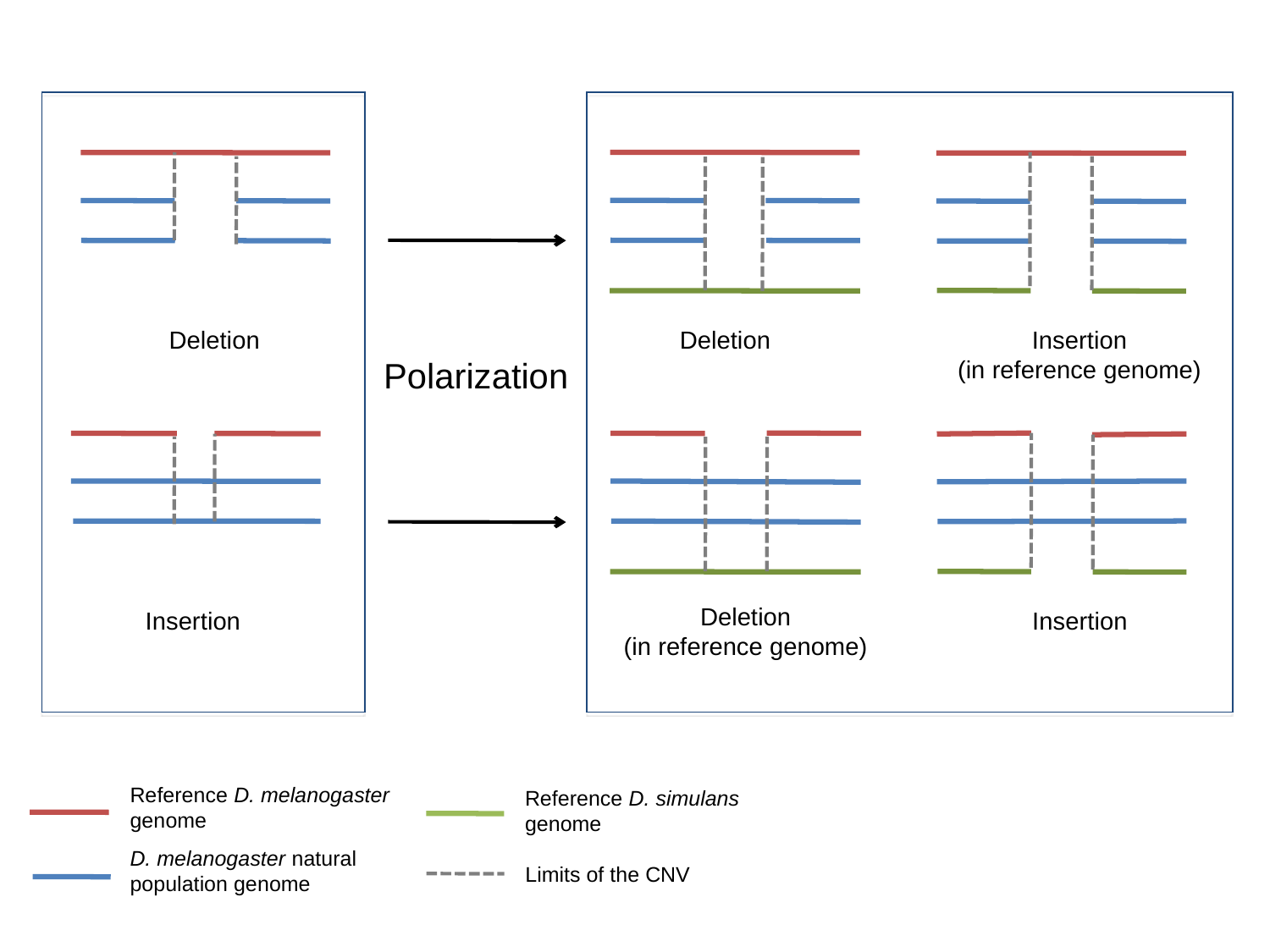

Deletion
Deletion
Insertion
(in reference genome)
Polarization
Deletion
(in reference genome)
Insertion
Insertion
Reference D. melanogaster genome
Reference D. simulans genome
D. melanogaster natural population genome
Limits of the CNV
